# Supplementary material for: Discrete LAT condensates encode antigen information from single pMHC:TCR binding events
Source: Nat Commun. 2022 Dec 2;13:7446. doi: 10.1038/s41467-022-35093-9 (PMC9718779; doi:10.1038/s41467-022-35093-9)
Supplement: Supplementary file 1 — Supplementary Information [file 41467_2022_35093_MOESM1_ESM.pdf]

Discrete LAT condensates encode antigen information from single pMHC:TCR binding events

Supplementary Information

Contents

Supplementary Figure 1 2
Supplementary Figure 2 4
Supplementary Discussion 1 6
Supplementary Figure 3 7
Supplementary Discussion 2 8
Supplementary Figure 4 9
Supplementary Figure 5 11
Supplementary Discussion 3 12
Supplementary References 12

Supplementary Figure 1

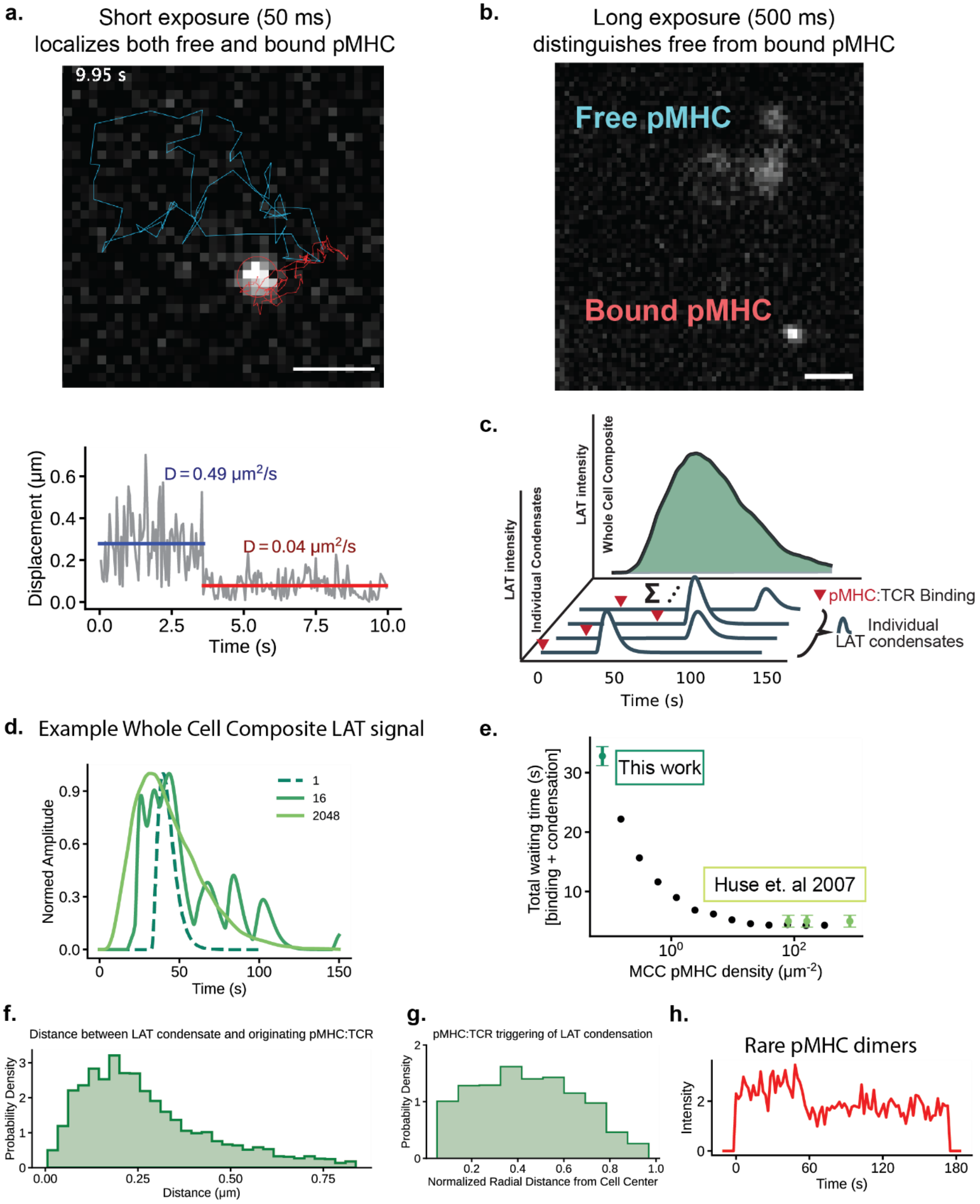

Supplementary Figure 1. Discrete LAT condensates produced by monomeric pMHC. a Top: TIRF image showing the full trajectory of a binding event. The MCC(Atto647) pMHC molecule was tracked with 50 ms camera exposure prior to binding (blue) and after binding (red). Bottom: Plot showing the displacement of the above pMHC through time. There is a sudden transition from a free state (blue) to a bound state (red) at 3.5 s, with an accompanying transition in the diffusion coefficient. **b** TIRF image using long camera exposure (500 ms) to distinguish bound pMHC from free pMHC. **c** Schematic showing the integrated LAT response at the cellular level under high stimulation conditions. **d** Example traces showing whole cell LAT response for different numbers of simulated LAT condensate pulses. The dashed line is the average expected single pulse: ≈ 7 s delay from initial cell-bilayer contact until the pMHC binds to the TCR, then a delay time of ≈ 25 s between binding and condensation, and then a LAT condensate lifetime with a lifetime of ≈ 25 s. The solid lines are generated from summing N randomly sampled LAT condensate pulses, renormalized such that the peak amplitude is 1. **e** Plot showing the simulated mean total offset time, from the moment of T cell exposure to pMHC ligand until detected LAT signal (across the whole cell). See “Simulation of Whole Cell Integrated LAT Signal” in Methods. The data point specified by “This work” derives from the sum of the estimated per pMHC on-rate with TCR and the per pMHC(TCR) delay time until LAT condensation, both of which are directly observed in this study (the mean and estimated error is derived from the distribution in main Fig. 4b). The data points specified by “Huse et al.” were adapted from Figure 3A in the referenced study. **f** Histogram of distances between the centroid of the LAT condensate and the centroid of its associated pMHC:TCR binding event. Distances are aggregated from all time frames in which both the condensate and its pMHC:TCR are present, yielding a temporally global distance histogram. **g** The probability of LAT condensation as a function of normalized radial distance $d = \frac{|\vec{r}|}{|\vec{r}|+|\vec{b}|}$, where $|\vec{r}|$ is the distance from the center of the cell to the point of condensation and $|\vec{b}|$ is the distance from the point of condensation to the nearest boundary point of the cell. The distribution was normalized to the area of each annulus corresponding to a particular radial bin. **h** Intensity trace showing the binding of a pMHC dimer. Intensity was normalized to a single step. This class of binding event was excluded from further analysis, i.e., only the effects of monomeric pMHC were studied in detail. Source data are provided as a Source Data file.

Supplementary Figure 2

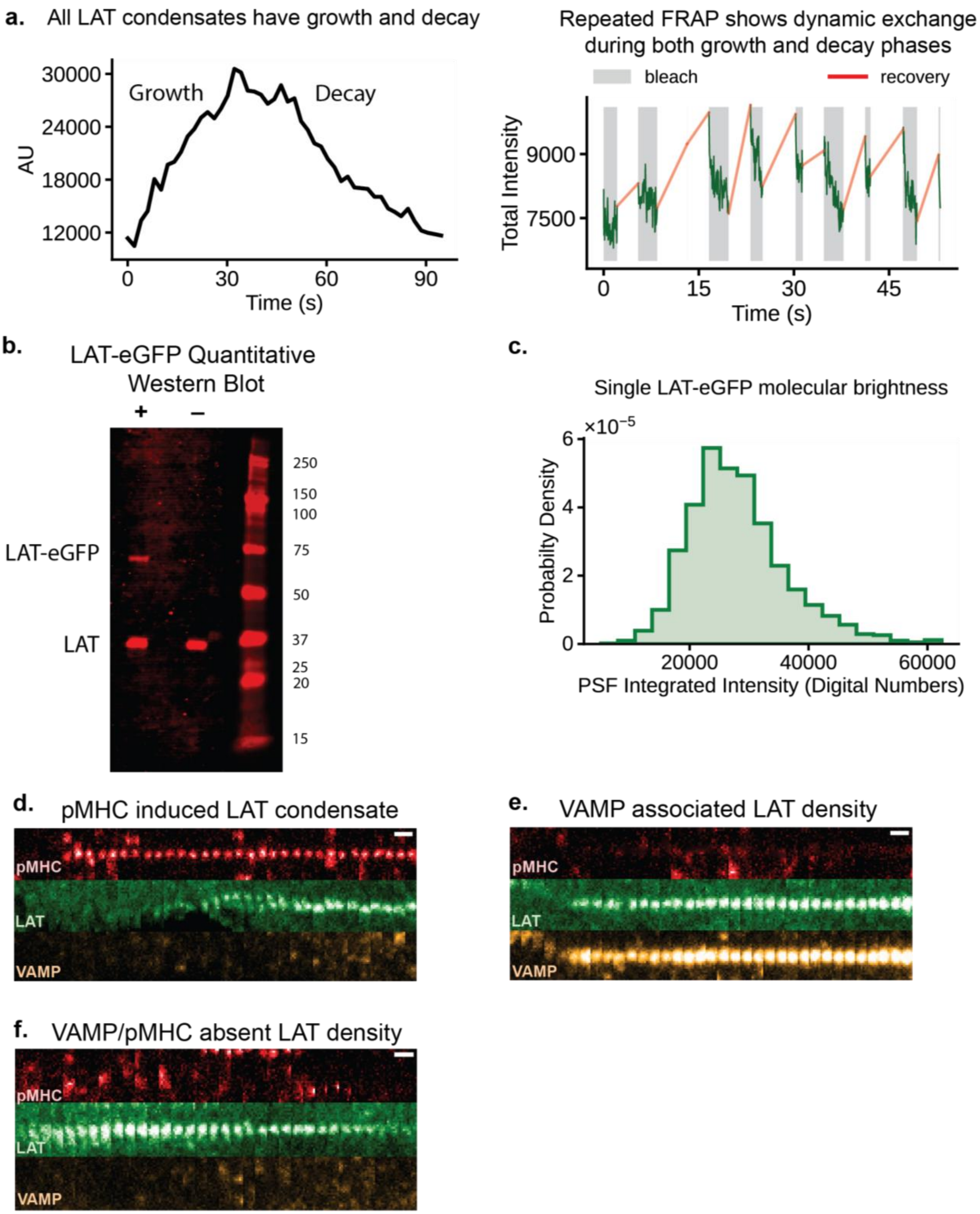

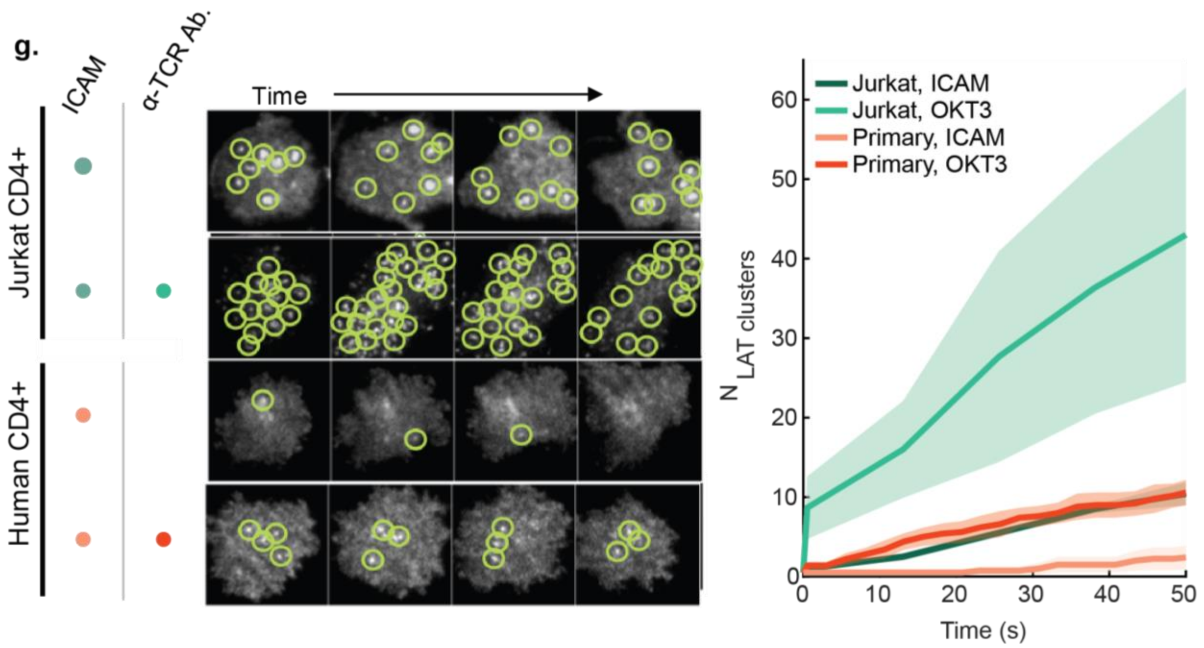

Supplementary Figure 2. Quantitation and composition of LAT condensates. **a** Left: Example time trace of the intensity of LAT condensate, showing the characteristic growth and decay phases intrinsic to all LAT condensates. Right: Periodic bleaching of a LAT condensate shows rapid recovery throughout the lifetime of the LAT condensate. **b** Quantitative western blot using a primary pan-LAT antibody and a secondary IR conjugated antibody. The + lane contained primary T cells transduced with MSCV plasmid containing LAT-eGFP, while the − lane contained untransduced primary T cells. Contrast and background subtraction have been applied to this image. FACS showed %15.2 of the cells in this batch were successfully transduced with LAT-eGFP. Representative from two experiments done. **c** Distribution of the integrated net intensity within spot detections of single LAT-eGFP molecules at the plasma membrane, used for quantitative fluorescence to determine the number of LAT molecules within LAT condensates; see Supplementary Movie 6. (**d-f**) Representative time-series of TIRF images of primary murine T cells expressing LAT-mScarleti and VAMP-mNeonGreen deposited onto supported membranes containing ICAM-1 and MCC(Atto647) pMHC:TCR. **d** Condensation of LAT in response to a pMHC:TCR binding event, but lacking any notable VAMP dynamics. **e** A LAT density associated with VAMP, but not MCC. **f** A LAT density either associated with dark MCC binding, or has an alternate origin, independent of VAMP and MCC. **g** Left: Time series TIRF images of LAT clustering seen in both Jurkat cells (top) and primary human T cells (bottom) under adhesion only (human his-ICAM) and adhesion + stimulation conditions (biotin-OKT3). Bilayers contained biotin-CAP-PE and Ni-NTA-DOGS lipids; and were coated with streptavidin and activated with Ni^{2+} to allow binding with biotin-OKT3 and his-ICAM-1, respectively. Right: plot showing the cumulative numbers of distinct LAT condensates seen through time. The mean was plotted as a solid dark line, while the lighter colored error bands were derived from ± 1 standard deviation. Compare to Figure 5. Source data are provided as a Source Data file.

Supplementary Discussion 1

Delivery of cytosolic LAT to the plasma membrane has been reported via VAMP7-associated vesicles [1,2]. To confirm the pMHC:TCR-induced condensates studied here are distinct, we performed experiments on T cells simultaneously expressing LAT-mScarleti and VAMP7-mNeonGreen. LAT condensates associated with pMHC:TCR binding events did not show any appreciable inclusion of VAMP7 (Supplementary Figure 2d). However, at later time points (>30 s after cell landing), some dense features of LAT did appear in the TIRF images that were not associated with pMHC:TCR and contained VAMP7 (Supplementary Figure 2e). Some LAT features were also observed that lacked VAMP7 and were not associated with detectable pMHC:TCR (Supplementary Figure 2f), possibly corresponding to pMHC:TCR-induced condensates in which the fluorescent label on pMHC has been bleached or some other form of LAT oligomerization [3]. In this study we focus on LAT condensates whose origin can be directly associated to a pMHC:TCR binding event.

Supplementary Figure 3

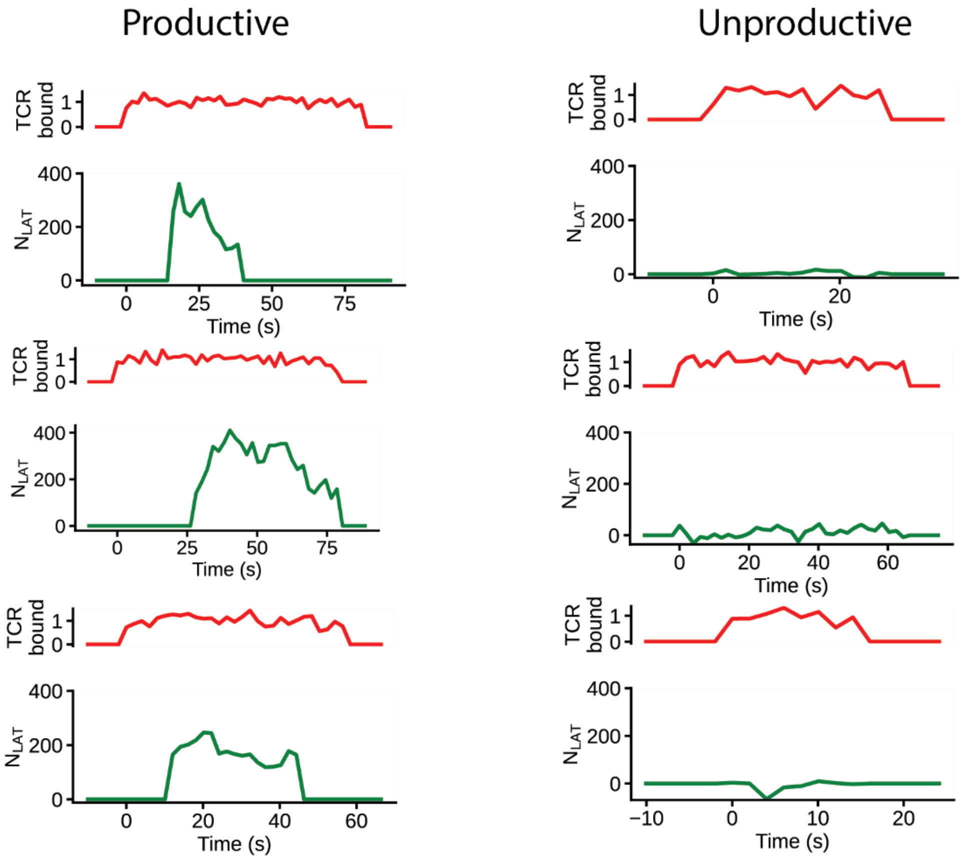

Supplementary Figure 3. Representative traces of monomeric pMHC:TCR binding and its associated LAT dynamics, compare to main Fig. 1h. Red traces are the normalized intensity of the bound pMHC. Green traces follow the local maxima of LAT intensity near the pMHC binding event, remapped to number of LAT (see main Fig. 2). Source data are provided as a Source Data file.

Supplementary Discussion 2

Plausible negative feedback mechanisms for how LAT condensates dissipate prior to pMHC:TCR unbinding include further LAT modification – e.g., ubiquitylation [4] – or ZAP-70 inactivation [5]. Both of these processes would occur locally with timescales set by individual LAT condensation or initial pMHC:TCR binding time. Additionally, once formed, pMHC:TCR complexes begin to move in a directed retrograde flow pattern towards the center of the immunological synapse. Early studies mapping geometric position within the immunological synapse to TCR signaling activity identified differential effects in the immunological synapse center favoring signal downregulation [6,7]. It is plausible that the prevailing cytoplasmic conditions in more central positions also disfavor LAT condensation and moving LAT condensates into this region might facilitate dispersal.

Supplementary Figure 4

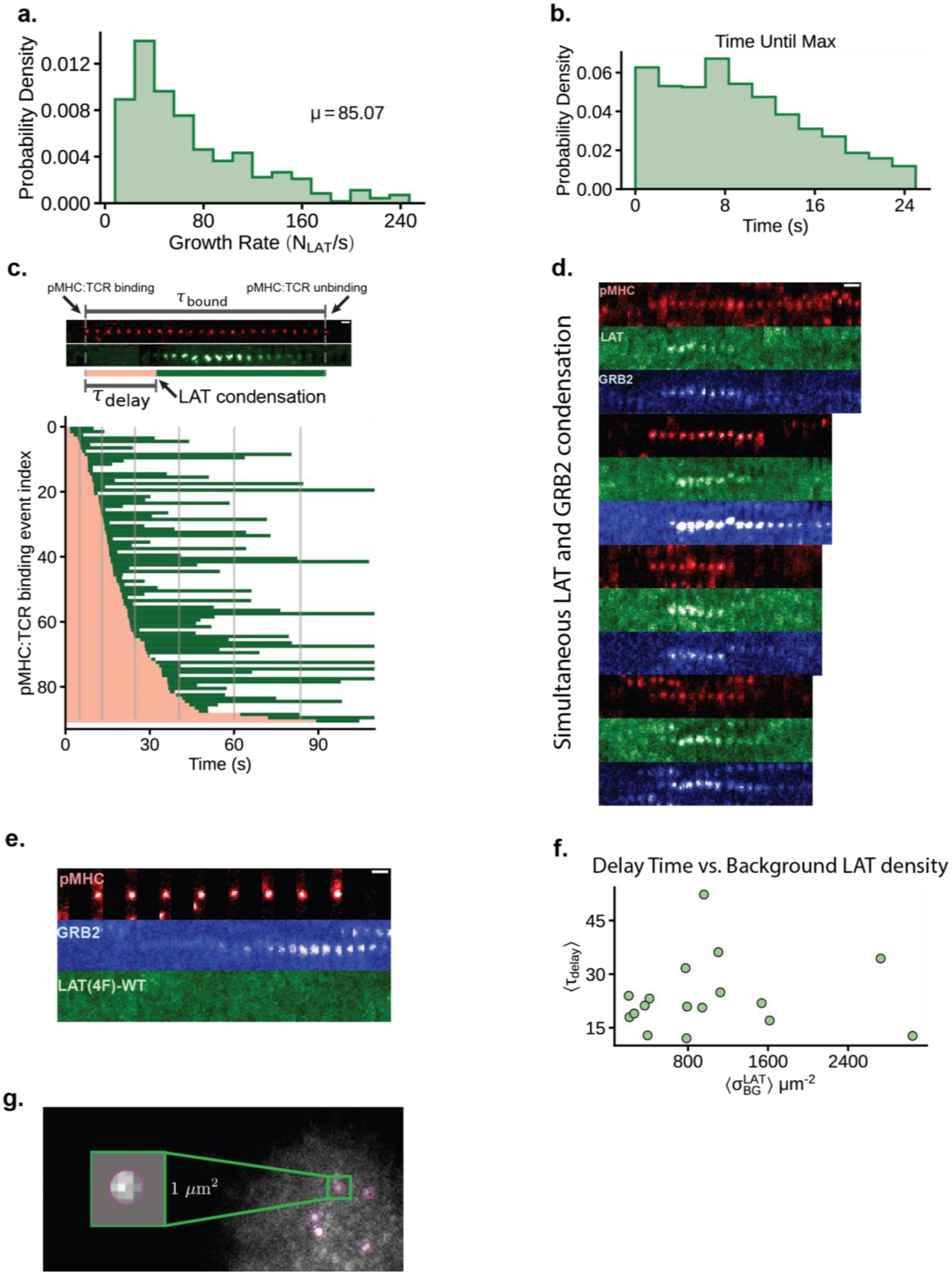

Supplementary Figure 4. a Histogram of the growth rate of LAT condensates within the first 4-6 s of detection. **b** Histogram of the time it takes for LAT condensates to reach peak net intensity. **c** Ensemble of 91 pMHC:TCR binding events that produce LAT condensates illustrating the distribution of LAT delay times. **d** More representative TIRF images of LAT-eGFP(P2A)GRB2-mCherry examples showing the strong coincidence of LAT condensation and GRB2 clustering. **e** Representative trace of a cell expressing LAT(4F)-eGFP(P2A)GRB2-mCherry showing that the LAT(4F) construct fails to participate in pMHC triggered clustering events. **f** Scatter plot of the mean LAT condensation delay time observed in a cell against the mean background LAT density within the same cell. Each point is a cell average. **g** Image illustrating the sampling of mNeonGreen-Grb2 background and the comparison of the mean internal intensity of a spot detection and the mean external intensity of the local background. Source data are provided as a Source Data file.

Supplementary Figure 5

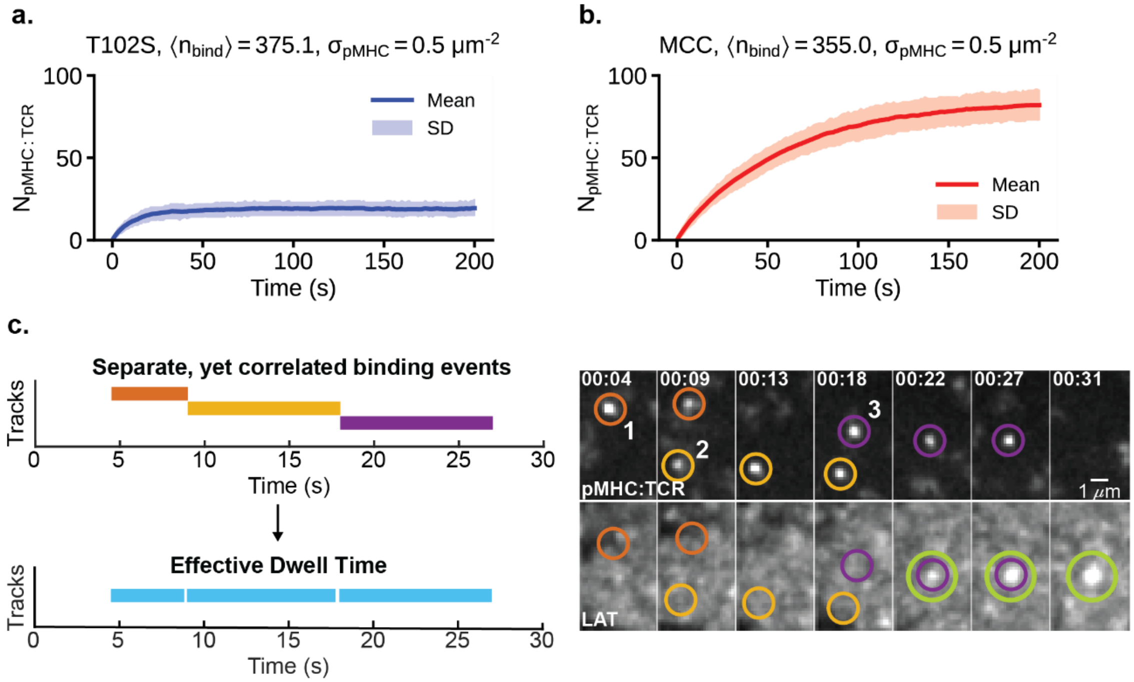

Supplementary Figure 5. a Plot of the number of pMHC:TCR complexes through time for T102S pMHC. From 250 Gillespie simulated trajectories: the mean along with the middle 68% of trajectories (SD) were plotted. **b** Plot of the number of pMHC:TCR complexes through time for MCC pMHC. From 250 Gillespie simulated trajectories: the mean along with the middle 68% of trajectories (SD) were plotted. See Methods for more details. **c** Left: schematic of how distinct binding events, that are locally coincident, may potentiate LAT condensation. Right: TIRF images showing rapid LAT condensation after a series a locally coincident T102S(Atto647) pMHC:TCR binding events. Source data are provided as a Source Data file.

Supplementary Discussion 3

Supplementary Figure 5c illustrates an example of spatiotemporally correlated T102S pMHC:TCR binding events that appear to nucleate a single LAT condensate. Evidence of such spatiotemporal correlations enhancing the potency of short dwell time ligands has been detected previously in experiments mapping pMHC:TCR binding events to T cell activation [8]. Such effects may also have contributed to earlier observations of apparent pMHC:TCR cooperativity at pMHC levels so low that multiple pMHC:TCR complexes were unlikely to coexist at the same time [9]. Here we present visualization data suggesting that the LAT condensation phase transition itself may be an integrating mechanism capable of establishing cooperativity between pMHC:TCR complexes that do not exist simultaneously.

Supplementary References

1. Balagopalan, L. *et al.* Plasma membrane LAT activation precedes vesicular recruitment defining two phases of early T-cell activation. *Nat Commun* 9, 2013 (2018).

2. Soares, H. *et al.* Regulated vesicle fusion generates signaling nanoterritories that control T cell activation at the immunological synapse. *J Exp Med* 210, 2415–2433 (2013).

3. Raab, M. *et al.* LFA-1 activates focal adhesion kinases FAK1/PYK2 to generate LAT-GRB2-SKAP1 complexes that terminate T-cell conjugate formation. *Nat Commun* 8, 16001 (2017).

4. Balagopalan, L. *et al.* c-Cbl-Mediated Regulation of LAT-Nucleated Signaling Complexes ▿ †. *Mol Cell Biol* 27, 8622–8636 (2007).

5. Sjölin-Goodfellow, H. *et al.* The catalytic activity of the kinase ZAP-70 mediates basal signaling and negative feedback of the T cell receptor pathway. *Sci Signal* 8, ra49–ra49 (2015).

6. Mossman, K. D., Campi, G., Groves, J. T. & Dustin, M. L. Altered TCR Signaling from Geometrically Repatterned Immunological Synapses. *Science* 310, 1191–1193 (2005).

7. Lee, K.-H. *et al.* The Immunological Synapse Balances T Cell Receptor Signaling and Degradation. *Science* 302, 1218–1222 (2003).

8. Lin, J. J. Y. *et al.* Mapping the stochastic sequence of individual ligand-receptor binding events to cellular activation: T cells act on the rare events. *Sci Signal* 12, eaat8715 (2019).

9. Manz, B. N., Jackson, B. L., Petit, R. S., Dustin, M. L. & Groves, J. T-cell triggering thresholds are modulated by the number of antigen within individual T-cell receptor clusters. *Proc National Acad Sci* 108, 9089–9094 (2011).

10. Bridgeman, J. S., Sewell, A. K., Miles, J. J., Price, D. A. & Cole, D. K. Structural and biophysical determinants of αβ T-cell antigen recognition. *Immunology* 135, 9–18 (2012).

11. Stepanek, O. *et al.* Coreceptor Scanning by the T Cell Receptor Provides a Mechanism for T Cell Tolerance. *Cell* 159, 333–345 (2014).
