# Supplementary material for: Discrete LAT condensates encode antigen information from single pMHC:TCR binding events
Source: Nat Commun. 2022 Dec 2;13:7446. doi: 10.1038/s41467-022-35093-9 (PMC9718779; doi:10.1038/s41467-022-35093-9)
Supplement: Supplementary file 3 — Description of Additional Supplementary Files [file 41467_2022_35093_MOESM3_ESM.pdf]

Description of Additional Supplementary Files

Title: **Supplementary Movie 1**
Description: Histidine tagged MHC loaded with MCC(C) peptide labelled with Atto647 diffuses on an Ni-NTA lipid bilayer and transitions to a bound state underneath a T cell. ICAM-1 is present for cellular adhesion.

Title: **Supplementary Movie 2**
Description: A primary mouse T cell expressing TCR(AND) and LAT-eGFP is deposited onto a bilayer containing $15.9\ \mu m^{-2}$ of MCC pMHC. ICAM-1 is present for T cell adhesion.

Title: **Supplementary Movie 3**
Description: A primary mouse T cell expressing TCR(AND) and LAT-eGFP is deposited onto a bilayer containing $0.43\ \mu m^{-2}$ of MCC pMHC. ICAM-1 is present for T cell adhesion.

Title: **Supplementary Movie 4**
Description: Zoomed in example of LAT condensation near a pMHC:TCR binding event. A primary mouse T cell expressing TCR(AND) and LAT-eGFP is deposited onto a bilayer containing MCC(Atto647) pMHC. Using a camera exposure of 500 ms on the 640 nm channel, bound pMHC were selectively visualized. LAT (green) is shown to condense near a MCC pMHC (red) binding event.

Title: **Supplementary Movie 5**
Description: Cell wide behavior of LAT condensation relative to pMHC:TCR binding events. A primary mouse T cell expressing TCR(AND) and LAT-eGFP is deposited onto a bilayer containing MCC(Atto647) pMHC. Using a camera exposure of 500 ms on the 640 nm channel, bound pMHC were selectively visualized. LAT (green) is shown to condense near MCC pMHC (red) binding events.

Title: **Supplementary Movie 6**
Description: Example of a cell with low expression of LAT-eGFP. A primary mouse T cell expressing LAT-eGFP was imaged continuously with 50 ms camera exposure. Spots that underwent single step photobleaching were used to construct a molecular brightness histogram.

Title: **Supplementary Movie 7**
Description: An example image of LAT-mCherry condensation. The condensation behavior showed no noticeable qualitative difference from LAT-eGFP. Primary mouse CD4+ T cells transduced with LAT-mCherry were deposited onto supported lipid bilayer with ICAM and MCC(Atto647N)-MHC ($0.56\ \mu m^{-2}$). LAT-mCherry condensation was observed over time with TIRF microscopy (560 nm excitation). Note that this measurement was conducted with calcium-responsive dye for another experiment (data not shown), but LAT-mCherry behavior was not affected by the dye.

Title: **Supplementary Movie 8**

Description: Multichannel monitoring of MCC pMHC (red, left), LAT-eGFP (green, middle), and NFAT (pink, right) within a primary mouse T cell. T cells expressing LAT(G135D)-eGFP were deposited onto bilayers containing MCC(Atto647) pMHC and ICAM-1.
