# Supplementary material for: Discrete LAT condensates encode antigen information from single pMHC:TCR binding events
Source: Nat Commun. 2022 Dec 2;13:7446. doi: 10.1038/s41467-022-35093-9 (PMC9718779; doi:10.1038/s41467-022-35093-9)
Supplement: Supplementary file 12 — Reporting Summary [file 41467_2022_35093_MOESM12_ESM.pdf]

nature portfolio

Corresponding author(s): Jay T. Groves, Darren B. McAffee

Last updated by author(s): September 16th, 2022

Reporting Summary

Nature Portfolio wishes to improve the reproducibility of the work that we publish. This form provides structure for consistency and transparency in reporting. For further information on Nature Portfolio policies, see our Editorial Policies and the Editorial Policy Checklist.

Statistics

For all statistical analyses, confirm that the following items are present in the figure legend, table legend, main text, or Methods section.

n/a	Confirmed	
☐	☒	The exact sample size (*n*) for each experimental group/condition, given as a discrete number and unit of measurement
☐	☒	A statement on whether measurements were taken from distinct samples or whether the same sample was measured repeatedly
☐	☒	The statistical test(s) used AND whether they are one- or two-sided<br>*Only common tests should be described solely by name; describe more complex techniques in the Methods section.*
☒	☐	A description of all covariates tested
☒	☐	A description of any assumptions or corrections, such as tests of normality and adjustment for multiple comparisons
☐	☒	A full description of the statistical parameters including central tendency (e.g. means) or other basic estimates (e.g. regression coefficient) AND variation (e.g. standard deviation) or associated estimates of uncertainty (e.g. confidence intervals)
☐	☒	For null hypothesis testing, the test statistic (e.g. *F*, *t*, *r*) with confidence intervals, effect sizes, degrees of freedom and *P* value noted<br>*Give P values as exact values whenever suitable.*
☒	☐	For Bayesian analysis, information on the choice of priors and Markov chain Monte Carlo settings
☒	☐	For hierarchical and complex designs, identification of the appropriate level for tests and full reporting of outcomes
☒	☐	Estimates of effect sizes (e.g. Cohen's *d*, Pearson's *r*), indicating how they were calculated

Our web collection on statistics for biologists contains articles on many of the points above.

Software and code

Policy information about availability of computer code

Data collection	Micro-manager (1.4.22)
Data analysis	Fiji (on ImageJ 1.53f51)<br>TrackMate (7.6.1)<br>The custom code and python package environment is available from: https://doi.org/10.5281/zenodo.7199954

For manuscripts utilizing custom algorithms or software that are central to the research but not yet described in published literature, software must be made available to editors and reviewers. We strongly encourage code deposition in a community repository (e.g. GitHub). See the Nature Portfolio guidelines for submitting code & software for further information.

Data

Policy information about availability of data

All manuscripts must include a data availability statement. This statement should provide the following information, where applicable:

- Accession codes, unique identifiers, or web links for publicly available datasets
- A description of any restrictions on data availability
- For clinical datasets or third party data, please ensure that the statement adheres to our policy

The dataset of microscopy acquisitions and other supporting data are available from https://doi.org/10.5281/zenodo.7199954

Human research participants

Policy information about studies involving human research participants and Sex and Gender in Research.

Reporting on sex and gender	N/A
Population characteristics	N/A
Recruitment	N/A
Ethics oversight	N/A

Note that full information on the approval of the study protocol must also be provided in the manuscript.

Field-specific reporting

Please select the one below that is the best fit for your research. If you are not sure, read the appropriate sections before making your selection.

☒ Life sciences ☐ Behavioural & social sciences ☐ Ecological, evolutionary & environmental sciences

For a reference copy of the document with all sections, see nature.com/documents/nr-reporting-summary-flat.pdf

Life sciences study design

All studies must disclose on these points even when the disclosure is negative.

Sample size	Lymph node and spleen samples were gathered from mice to determine various molecular properties (kinetics and stoichiometries within cells). As such, sampling of mice was performed until such quantities could be estimated, and sufficiency was gauged by the quality of the distributions. For imaging experiments, 30-60 million T cells would be prepared with transduction. Approximately 1-2 million cells per imaging chamber were used in order to catch T cells landing on the bilayer, but not have the slide overcrowded. Each result obtained from these samples was repeated from at least three separate mice.
Data exclusions	Mice older than 30 weeks were excluded as they generally had reduced transduction efficiencies. T cells that lacked transduction were excluded (unless specified as a negative control). T cells with abnormal phenotypes (e.g., failure to crawl on ICAM-1 bilayers) were excluded. pMHC:TCR binding events that merged (i.e., could not optically be resolved spatially from other binding events) were excluded.
Replication	The results in this study were reproducible. Broadly, the results within this study were observed across a minimum of 3 mice, and upwards of 20 mice for some of the results. For experiments involving Jurkat cells, 3 separately thawed samples showed the similar results after various passages.
Randomization	There were no relevant covariates in this study. This study was not designed to reject a particular null-hypothesis, but instead gather evidence, observations, and to quantify new cellular phenomena across an entire population.
Blinding	Blinding was not relevant in this study. This study was not designed to reject a particular null-hypothesis, but instead gather evidence, observations, and to quantify new cellular phenomena across an entire population.

Reporting for specific materials, systems and methods

We require information from authors about some types of materials, experimental systems and methods used in many studies. Here, indicate whether each material, system or method listed is relevant to your study. If you are not sure if a list item applies to your research, read the appropriate section before selecting a response.

Materials & experimental systems

n/a	Involved in the study	
☐	☒	Antibodies
☐	☒	Eukaryotic cell lines
☒	☐	Palaeontology and archaeology
☐	☒	Animals and other organisms
☒	☐	Clinical data
☒	☐	Dual use research of concern

Methods

n/a	Involved in the study	
☒	☐	ChIP-seq
☒	☐	Flow cytometry
☒	☐	MRI-based neuroimaging

Antibodies

Antibodies used	LAT (E3U6J) XP® Rabbit mAb; Cell Signaling Technology (45533)<br>IRDye® 680RD Donkey anti-Rabbit IgG; Li-cor (926-68073)<br>Biotin anti-human CD3 (OKT3) antibody; BioLegend (317319)<br>All primary antibodies were used at 1:1000 dilution unless otherwise stated.
Validation	Each vendor for the antibodies above quality control tested the antibodies against targets by immunofluorescent staining with flow cytometric analysis and western blot analysis.

Eukaryotic cell lines

Policy information about cell lines and Sex and Gender in Research

Cell line source(s)	Parental Jurkats (E6-1), a gift from Art Weiss Laboratory.
Authentication	Identity of Jurkat is routinely validated using an anti-TCR Vbeta mAb (C305) generated by Dr. Weiss.
Mycoplasma contamination	The parental Jurkat line had been tested mycoplasma negative in the recent few years prior to our acquisition.
Commonly misidentified lines (See ICLAC register)	The Jurkat cell line was not among the misidentified cell lines.

Animals and other research organisms

Policy information about studies involving animals; ARRIVE guidelines recommended for reporting animal research, and Sex and Gender in Research

Laboratory animals	Mus musculus; B10.Cg-Tg(TcrAND)53Hed/J; Jackson Laboratory; 002761<br>Mus musculus; B10.BR-H2k2 H2-T18a/SgSnJ; Jackson Laboratory; 000465
Wild animals	N/A
Reporting on sex	Both male and female mice were used. No differences were observed.
Field-collected samples	N/A
Ethics oversight	All animal work was performed with prior approval by the IACUC committee, Lawrence Berkeley National Laboratory Animal Welfare and Research Committee, under the approved protocol 17703.

Note that full information on the approval of the study protocol must also be provided in the manuscript.
